# Performance of Detecting IgM Antibodies against Enterovirus 71 for Early Diagnosis

**DOI:** 10.1371/journal.pone.0011388

**Published:** 2010-06-30

**Authors:** Feihai Xu, Qiang Yan, Hua Wang, Jianjun Niu, Liang Li, Fengcai Zhu, Shuizhen He, Shiyin Zhang, Zuxing Weng, Tong Cheng, Yijun Cai, Delei He, Yixin Chen, Shengxiang Ge, Anthony E. T. Yeo, Jun Zhang, Mun-Hon Ng, Ningshao Xia

**Affiliations:** 1 National Institute of Diagnostics and Vaccine Development in Infectious Disease, Xiamen University, Xiamen, China; 2 Jiangsu Provincial Center for Disease Control and Prevention, Nanjing, China; 3 Xiamen Center for Disease Control and Prevention, Xiamen, China; Cochrane Acute Respiratory Infections Group, Italy

## Abstract

Enterovirus 71 (EV71) infection is more likely to induce severe complications and mortality than other enteroviruses. Methods for detection of IgM antibody against EV71 had been established for years, however, the performance of the methods in the very early diagnosis of EV71 infection had not been fully evaluated, which is especially meaningful because of the short incubation period of EV71 infection. In this report, the performance of an IgM anti-EV71 assay was evaluated using acute sera collected from 165 EV71 infected patients, 165 patients infected with other enteroviruses, and more than 2,000 sera from healthy children or children with other infected diseases. The results showed a 90% sensitivity in 20 patients who were in their first illness day, and similar sensitivity remained till 4 days after onset. After then the sensitivity increased to 95% to 100% for more than one month. The specificity of the assay in non-HFMD children is 99.1% (95% CI: 98.6–99.4), similar as the 99.9% specificity in healthy adults. The cross-reaction rate in patients infected with other non-EV71 enteroviruses was 11.4%. In conclusion, the data here presented show that the detection of IgM anti-EV71 by ELISA affords a reliable, convenient, and prompt diagnosis of EV71 infection.

## Introduction

Enterovirus 71 (EV71) and coxsackievirus A16 (CA16) are the principal pathogens of hand foot and mouth disease (HFMD). EV71 is of special concern because it is more likely to induce severe complications and mortality than other enteroviruses, and has become endemic in Southeast Asia for tens of years [1], [2]. It has caused several wide spread epidemics in this region since 1997 and is expected to continue to do so in the future [3]–[6]. There is no effective anti-virus treatment for EV71 and control depends on prompt diagnosis and timely implementation of appropriate measures to contain the spread of the infection [7], [8].

Laboratory diagnosis of EV71 relies mainly on detection of the viral genome by reverse transcription polymerase chain reaction or on virus isolation techniques [9]–[13]. However, these methods were unaffordable in most community clinics in developing countries in which most epidemics occurred. Tsao et al. (2002) showed and confirmed later by Wang et al. (2004) that IgM anti-EV71 was detectible in patients [14], [15]. However, due to the very limited number of evaluated clinical samples in these studies, the diagnosis accuracy of IgM anti-EV71 test had not been well determined [16]. The aim of this study was to assess the performance of detecting IgM anti-EV71 for early diagnosis of patients with HFMD.

## Materials and Methods

### Ethic Statement

Written informed consent was obtained from each subject. Independent Ethics Committee approval was obtained from the Ethics Committee of the National Institute of Diagnostics and Vaccine Development in infectious diseases.

### Study design

The sensitivity of the IgM anti-EV71 assay was evaluated in HFMD patients who were confirmed to be recently EV71 infection, and was classified by the days apart from the onset. The specificity of the assay was evaluated in children patients with confirming diagnosis of other respiratory diseases. The cross-reactivity of the assay was evaluated in HFMD patients infected by other enteroviruses.

### Serum samples

A total of 376 serum samples were collected from HFMD patients, herpangina, aseptic meningitis, or encephalitis between March and September 2008. Of these samples, 221 were collected from 165 EV71-infected patients with the mean age of 2.6±2.1, 155 were from CA16–infected patients with the mean age of 2.7±2.5. The infection of EV71 or CA16 among these patients was determined by detection of the viral RNA by reverse transcript PCR. Twelve serum samples collected from patients infected by other enteroviruses (4 coxsackievirus A2, 1 coxsackievirus A4, 1 coxsackievirus B3, 2 coxsackievirus B4, 2 coxsackievirus B5, and 2 echovirus 6) were gifts from Dr. P. J. Chen of National Taiwan University, which were determined by virus isolation.

Control samples for this study included three groups. The first group included 128 sera from children patients with the following clinical features: Pneumonia (83 cases), Bronchitis (18), acute upper respiratory infections (15), and Influenza (12). The second group included 1907 stored sera from healthy children who received health examinations in with the mean age of 2.1±2.7. The third group included 807 sera from healthy adult blood donors. The EV71 neutralizing antibody titers of all control samples were less than 1∶100. Twenty serum samples positive with rheumatoid factor were also used to evaluate the possible disturbance to IgM testing. All serum samples were kept in aliquots at −20°C until use.

### Viral RNA extraction and PCR amplification

Viral RNA was extracted from the clinical specimens using a QIAamp Mini viral RNA Extraction Kit (Qiagen). The primers used for RT-PCR are listed in Table 1. RT-PCR amplification was performed using AccessQuickTM RT-PCR kit (Promega). Conditions for RT-PCR amplification were: 45 min of reverse transcription at 45°C; 5 min denaturation at 94°C; 35 cycles of 95°C for 40 sec, 53°C for 40 sec, 72°C for 40 sec; and then a final elongation step of 72°C for 5 min. The second round amplification was performed in 25 µl volumes, which contains 2.5 µl 10x PCR reaction buffer, 1 µl 10 mM dNTP, 0.5 µl 10 mM each primer, 0.5 µl Taq (TaKaRa), 1 µl of the first-round PCR product, and 19 µl Nuclease-Free Water, under the same conditions as the first-round PCR, except reverse transcription step. The PCR products were examined by electrophoresis with a 3.0% agarose gel.

**Table 1:** List of primers designed for the specific amplification of EV71 and CA16.

Primer name	Sequences(5′-3′)	Position(nt)
1st set primer		
EV71-F1	5′-AGAGCATGATTGAGACACG -3′	2607-2627
EV71-R1	5′-RTCTTTCTCYTGYTTGTGTTC-3′	3083-3063
2nd set primer		
EV71-F2	5′-CRGGRTTAGTTGGAGAGATAG-3′	2686-2706
EV71-R2	5′-CGCAGGTGACATGAATGG-3′	3020-3003
1st set primer		
CA16-F1	5′-TGCAGACATGATTGACCAG-3′	2457-2475
CA16-R1	5′-TCCCTACTGTCCTAATGCTA-3′	3163-3144
2nd set primer		
CA16-F2	5′-TGTGTTGAACCAYCACTCC-3′	2649-2667
CA16-R2	5′-TAGGTAAACAACTCGCATTT-3′	2824-2805

### Virus Isolation

Clinical specimens (including throat swabs and rectal swabs) were submitted for virus isolation. Samples were inoculated into RD and human laryngeal carcinoma (Hep-2) cell cultures. Cultures that exhibited a characteristic enterovirus CPE were further evaluated by RT-PCR and sequencing.

### Neutralization test

Laboratory methods for measuring EV71 neutralizing antibody followed standard protocol for the neutralization test on microtiter plates [17], [18]. Serum specimens were serially diluted two-fold (from 8 to 2048) and mixed with equal volume of EV71 (100 TCID_50_/50 µl) at 37°C for 60 min. The mixtures were incubated in replicate microplate cultures of human embryo rhabdomyosarcoma (RD) cells. Cytopathic effects (CPE) were read under an inverted microscope after 2 to 7 days. Neutralizing antibody titer was defined as the highest dilution of serum that prevented the occurrence of CPE.

### Detection of IgM anti-EV71

IgM anti-EV71 was detected using an IgM μ-chain capture enzyme-linked immunoabsorbant assay (ELISA) (Beijing Wantai Biological Pharmacy Enterprise Co., Ltd., China) according to manufacturer’s instruction. Briefly, 100 µl of dilution buffer were added to microplate wells pre-coated with anti-human IgM μ-chain, then 10 µl of serum samples were added and mixed tenderly, incubated at 37°C for 30 min, washed 5 times with washing buffer. Then 50 µl of the antigen solution, prepared by inactivating and fracturing of the cell free culture supernatant of an EV71 isolate JS/52-3/06, was added followed by 50 µl of a horseradish peroxidase (HRP)-conjugated mouse anti-EV71 monoclonal antibody, mixed, incubated for 30 min and then washed 5 times. 100 µl of TMB substrate was added then incubated at 37°C for 15 min before adding 50 µl 2N H_2_SO_4_ for terminating the reaction. The optical density (OD) was read at a wavelength of 450 nm with a reference filter of 620 nm. The cutoff value was calculated as 0.1+ mean OD value of the negative control. If the mean absorbance value of the negative control was lower than 0.05, this was treated as 0.05. Levels of anti-EV71 IgM was expressed in S/CO value, which was calculated as the ratio of the OD value obtained with the test sample to the cutoff value determined concurrently. A S/CO value not less than 1.0 indicated a positive result.

### Statistical analysis

The detection rate was compared between groups used two-sided Fishers exact test and 95% confidence intervals (CI) were calculated with the use of SPSS program (v. 11.5).

## Results

Table 2 compared the prevalence and mean levels of IgM anti-EV71 in serum samples collected from 165 EV71 infected patients at different times from 1 to 41 days after onset. The detection rate of IgM reached to 90% (18/20) in samples collected on the first day after onset, remained on this high level during the very early acute phase before the forth days after onset, and then increased to 95% to 100% for more than one month. The mean sensitivity of IgM test in the acute phase is 94.1% (95% CI: 90.2–96.8). The dynamic of IgM antibody level during acute phase was in consistent with the detection rate, as indicated by the S/CO values.

**Table 2:** Sensitivity of IgM anti-EV71 test at Different Times After Symptomatic Onset.

Days after onset	Tested No.	Positive No,	Rate (%) (95% CI)	Mean S/CO (SD)
1	20	18	90.0 (68.3–98.8)	4.66 (4.79)
2	25	22	88.0 (68.8–97.5)	6.00 (5.86)
3∼4	43	39	90.7 (77.9–97.4)	6.90 (4.63)
5∼10	53	51	96.2 (87.0–99.5)	11.92 (5.98)
11∼20	43	42	97.7 (87.7–99.9)	16.41 (6.05)
21–30	13	13	100.0 (83.2–100.0)	9.39 (6.17)
31–41	20	19	95.0 (78.9–99.9)	5.95 (4.37)
Total	221	208	94.1 (90.2–96.8)	9.78 (6.84)

Table 3 compared the occurrence of IgM anti-EV71 in serum samples obtained from other enterovirus-infected patients or control subjects. IgM anti-EV71 was detected in 19 of 155 samples (12.3%) from CA16-infected patients which indicated the cross-reaction and 0 of 12 samples from patients infected with other enteroviruses. The mean S/CO value of the cross-reaction samples was 2.0±1.4, which was significantly lower than that in positive samples from EV71-infected patients (mean  = 10.8±7.3). Among control subjects who were not infected by enteroviruses, the false positive results were found in 19 of 1907 samples (1.0%) from healthy children and 1 of 807 samples (0.12%) from healthy adults and absent in samples from patients with other childhood illnesses and the rheumatoid factor positive patients. The specificity of the assay was 98.7% (2990/3029, 95% CI: 98.2–99.1). Rheumatoid factor was not a confounder.

**Table 3:** Specificity and cross-reactivity of IgM anti-EV71 in control subjects.

Ctrl Subjects	No. tested	No. positive	Specificity (%)(95%CI)
Enterovirus infected children	167	19	88.6 (82.8–93.0)
CA16	153	19	87.6 (81.3–92.4)
Other enteroviruses	12	0	100 (73.5–100)
Other Children	2035	19	99.1 (98.6–99.4)
Other infectious diseases	128	0	100 (97.2–100)
Healthy children	1907	19	2 99.0 (98.5–99.4)
Healthy adults	807	1	99.9 (99.3–100)
Rheumatoid factor (+)	20	0	100 (83.2–100)

## Discussion

Several countries in Asia have instituted surveillance to provide early warning of outbreaks, which are largely depended on the timely case reports with pathogenic diagnosis from community clinics [19], [20]. Traditional laboratory diagnosis for EV71 is by cell culture followed by neutralization tests with serotype-specific antisera [9]. However, it requires weeks to obtain results. Consequently, molecular methods such as PCR have been developed to detect EV71 [10]–[13]. Unfortunately, these methods require expensive and specialized equipment and trained personnel, and can not be applied in most community clinics in developing world. IgM assays based on enzyme immunological techniques had been generally used for tens of years in most community clinics in many developing countries. The in-house IgM assays for diagnosis of acute EV71 infection had been established for years. However, the performance of the assays on early diagnosis of EV71 infected patients had not been fully evaluated. The results in this study showed a 90% sensitivity in patients who were in their first illness day, and similar sensitivity remained till 4 days after onset. After then the sensitivity increased to 95% to 100% for more than one month. Generally this sensitivity satisfies most of the demands for clinical early diagnosis as well as for early warning of outbreaks. The specificity of the assay in non-HFMD children is 99.1% (95% CI: 98.6–99.4), similar as the 99.9% specificity in healthy adults. It is noticed that substantial proportion (11.4%, 95%CI: 7.0–17.2) of children infected with other non-EV71 enteroviruses were positive by the IgM anti-EV71 assay. This cross-reaction was supposed to be due to the common epitopes among enteroviruses, and was mild as suggested by the lower S/CO values and the lower occurrence rate.

In conclusion, the data here presented show that the detection of IgM anti-EV71 by ELISA affords a reliable, convenient, and prompt diagnosis of EV71 infection. The whole assay takes 90 min using readily available ELISA equipment, is easy to perform with low cost, which made it suitable in clinical diagnosis as well as in public health utility.
